# Targeted Mutation of *NGN3* Gene Disrupts Pancreatic Endocrine Cell Development in Pigs

**DOI:** 10.1038/s41598-018-22050-0

**Published:** 2018-02-26

**Authors:** Timothy P. Sheets, Ki-Eun Park, Chi-Hun Park, Steven M. Swift, Anne Powell, David M. Donovan, Bhanu P. Telugu

**Affiliations:** 1 Department of Animal and Avian Sciences, University of Maryland, College Park, MD 20742 USA; 2 Animal Bioscience and Biotechnology Laboratory, USDA, ARS, Beltsville, MD 20705 USA; 3 RenOVAte Biosciences Inc, Reisterstown, MD 21136 USA

## Abstract

The domestic pig is an attractive model for biomedical research because of similarities in anatomy and physiology to humans. However, key gaps remain in our understanding of the role of developmental genes in pig, limiting its full potential. In this publication, the role of *NEUROGENIN 3* (*NGN3*), a transcription factor involved in endocrine pancreas development has been investigated by CRISPR/Cas9 gene ablation. Precomplexed Cas9 ribonucleoproteins targeting *NGN3* were injected into *in vivo* derived porcine embryos, and transferred into surrogate females. On day 60 of pregnancy, nine fetuses were collected for genotypic and phenotypic analysis. One of the piglets was identified as an in-frame biallelic knockout (Δ2/Δ2), which showed a loss of putative NGN3-downstream target genes: *NEUROD1* and *PAX4*, as well as insulin, glucagon, somatostatin and pancreatic polypeptide-Y. Fibroblasts from this fetus were used in somatic cell nuclear transfer to generate clonal animals to qualify the effect of mutation on embryonic lethality. Three live piglets were born, received colostrum and suckled normally, but experienced extreme weight loss over a 24 to 36-hour period requiring humane euthanasia. Expression of pancreatic endocrine hormones: insulin, glucagon, and somatostatin were lost. The data support a critical role of *NGN3* in porcine endocrine pancreas development.

## Introduction

### Development of pancreas and role of Ngn3: Lessons from mouse

Diabetes is a world-wide epidemic characterized by an imbalance in glucose homeostasis. Clinically, diabetes is caused by a loss of insulin (INS) production/secretion from pancreatic beta cells, or a decreased sensitivity to circulating INS. Among the available therapeutic options, cellular replacement therapy using isolated islets is widely used as a means for achieving balanced blood glucose levels^1,2^. *In vitro* differentiation of embryonic stem cells, induced pluripotent stem cells, and primary cells for the generation of functional glucose responsive INS producing cell types offer another promising alternative^3–5^. A complete understanding of the functionality of the regulatory factors during pancreas development is key to controlling the developmental program during *in vitro* differentiation, and is essential for generating functional beta-cells for replacement therapy^4^.

Our fundamental understanding of pancreatic development and the role of key genes comes from studies in mice. Pancreatic development in mice is tightly regulated by a transcriptional cascade, with dorsal and ventral pancreatic buds emerging from the foregut around embryonic day (E) 8.5. By E9.5, endocrine differentiation in the pancreatic buds is initiated with the onset of *Ngn3 (Neurogenin 3)* expression. By E13.5, the dorsal and ventral buds fuse together. Individual endocrine cell types arise by E14.5, and proliferation of epithelial cells accompanied by epithelial branching results in the formation of acini and ducts. On E18.5, individual islet cells begin to aggregate, thereby spatially distinguishing endocrine cells from ducts^6,7^. The role of candidate genes in exocrine and endocrine pancreatic development comes from knockout (KO) studies in mice. Ablation of *Pdx1* (*pancreatic and duodenal homeobox 1*) gene resulted in pancreatic agenesis, *Ptf1a* (*pancreas specific transcription factor 1a*) in the loss of exocrine pancreas development, and *Ngn3* in the loss of endocrine pancreas development, respectively^8,9^. The complete loss of *Ngn3*, a basic helix-loop-helix transcription factor results in a lack of endocrine precursor cells formation, causing early postnatal death. Additionally, loss of *Neurod1 (neuronal differentiation 1*), a direct downstream target of *Ngn3* and additional downstream transcription factors of *Ngn3* such as *Nkx2.2*, and *Rfx6*, have similarly been shown to be critical for murine endocrine pancreas formation and function^10–17^. Cumulatively, results from these studies highlight the critical role of *Ngn3* in endocrine pancreas development.

### Role of NGN3 in human pancreatic endocrine development

In mouse, several experimental models of *in vivo* differentiation have been performed to exploit regenerative signaling, as illustrated by the partial duct ligation model, partial pancreatectomy, and chemical-mediated tissue injury models^18–22^. In these models, subpopulations of low level *Ngn3/Sox9*^+^ epithelial cells have been shown to give rise to an elevated level of *Ngn3* endocrine precursors^23,24^. In humans, corroborating studies in fetal pancreas explant cultures have revealed reactivation of *NGN3* during *in vitro* development of the endocrine pancreas^25,26^. *NGN3* is reactivated in human cultured exocrine tissue prepared from clinical islet preparations^24^. Other groups have shown competence of *NGN3* to drive endocrine fate arising from purified and cultured ductal cells^18,27^. *NGN3* has also been implicated in endocrine regenerative signaling in cultured exocrine tissue from clinical donor pancreas preparations^20,21,24,28^. Cumulatively, evidence exists to support a role for *NGN3* in cellular reprogramming, differentiation, and maintenance of fetal progenitors^29^.

### Pancreas development in pig: a “bridge model”

Although islet transplantation and other cellular therapies offer hope in treating diabetes, limitations exist, which include a limited supply of available donor organs^1^. While alternative sources of cell therapy are being explored exhaustively in the mouse and human^30,31^, pig islets, which are in abundant supply have long been favored as a xenotransplantation source in humans^32–34^. Pigs are an ideal model for xenotransplantation because of similarities to human anatomy (e.g. organ size and vessel diameter)^32,35^. This together with the advent of advanced genome editing techniques, and future potential for pig-human chimeras^36^, make the pig model a valuable tool in regenerative medicine. Development of the pancreas in pig is only partially understood. One study in the pig described co-expression of INS and glucagon (GCG), and/or INS and somatostatin (SST) from the same cell across different stages of development and demonstrated a lack of islet mantle structure *in utero*^37^. In the mouse, partial duct ligation, partial pancreatectomy, and chemical-mediated tissue injury have demonstrated acute reactivation of *Ngn3*^18,19^. These studies also highlight differences between mouse and humans in regenerative signaling based on the nature and extent of tissue damage^18^, much of which highlight a critical role for *Ngn3* during the regenerative process. Jennings et. al. described differences between human and mouse pancreas development, specifically temporal expression patterns for *Ngn3*^29^ necessitating investigation in alternative non-rodent models such as pig, which are physiologically and phylogenetically closer to humans. *Ngn3* is necessary and sufficient for endocrine pancreas formation in the mouse^38^, but the functionality in any other non-rodent model, in this case, pig pancreas development has not been investigated.

### Genetic modification in pigs

In pigs and other large domestic animals, the recent advances in genome editing technologies, specifically CRISPR/Cas9, have made sophisticated genetic modifications feasible and routine^39^. This technology requires the Cas9 nuclease enzyme alongside a 20-mer RNA “guide sequence” adjoining a 3-nucleotide PAM motif (“NGG” for *S. pyogenes*) homologous to the genomic target site. The guide sequence and Cas9 binding sequence can be prepared as a chimeric single guide RNA (sgRNA). The CRISPR reagents can be delivered as expression plasmids, *in vitro* transcribed RNA, or the commercially available Cas9 protein pre-complexed with sgRNA. When introduced into somatic cells or embryos, the CRISPRs can introduce double strand breaks (DSB) and facilitate targeted gene modifications. Direct microinjection of precomplexed CRISPR-reagents into early embryos has been shown to result in highly efficient genomic modifications in pigs^40^. The CRISPRs, similar to other editors, engineer a double strand break (DSB) in DNA, which is predominantly repaired by non-homologous end-joining (NHEJ) pathway. The NHEJ pathway has a high error rate and thus a high probability of creating errors in open reading frame (ORF), and hence a KO of the gene. Once a modification of interest is achieved, subsequent somatic cell nuclear transfer (SCNT) can be used to generate a cohort of animals harboring the desired targeted genome modifications. Using this experimental pipeline, we set to investigate the role of *NGN3* in a pig model. Our hypothesis is that loss of *NGN3* will result in the ablation of endocrine but not exocrine pancreas.

## Results

### Sequencing of porcine *NGN3*

**Figure 1:**
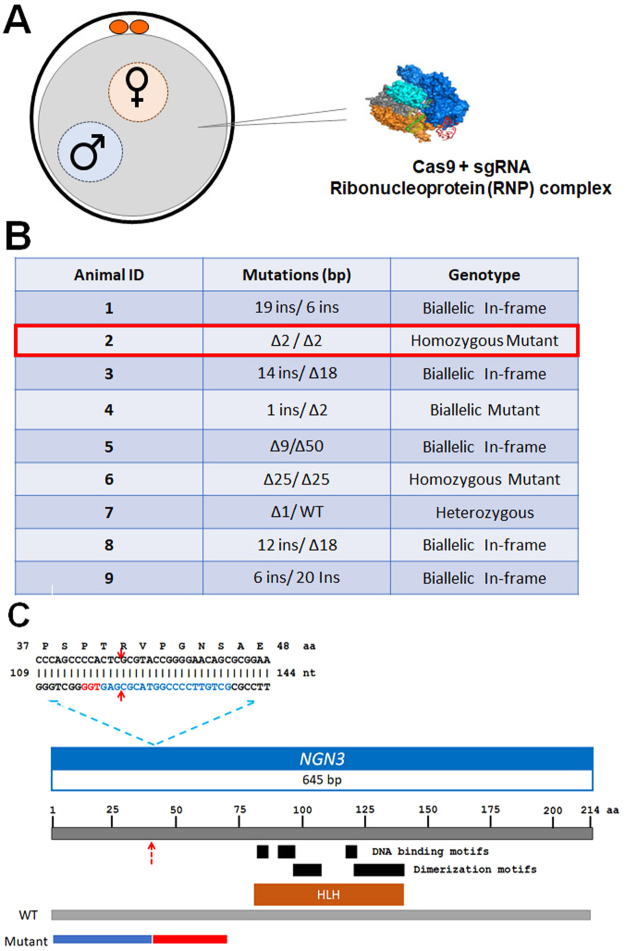
Generation of NGN3 edited porcine fetuses. (**A**) Illustration showing microinjection of Cas9 ribonucleoprotein into the cytoplasm of 1-cell zygote. (**B**) E60 fetal genotypes resulting from injection of CRISPR/Cas9/sgRNA complex. Columns from the top row across, include fetus identification number, change in sequence (number of base pairs inserted (ins) or deleted (∆)) compared to WT for allele 1 and allele 2, followed by genotype summary. (**C**) Graphic of the open reading frame for NGN3 identified in this study with a zoomed-in image of the CRISPR targeting site and Δ2/Δ2 mutation from Animal #2. Red arrows show the CRISPR cleavage site, PAM motif is indicated in red, sgRNA2 is shown in blue. Cleavage site (red arrow) occurs after the 40th codon. Schematic of the predicted conserved domains and binding sites from NCBI CD-Search program. Illustration comparing WT amino acid sequence to animal #2, where similar sequence is depicted in blue and the differences are shown in red resulting from the Δ2/Δ2 mutation. A deletion which causes a frameshift should effectively eliminate >80% of the protein.

The advances in genetic engineering technologies have brought genome editing in pigs to the forefront. That said, difficulties still remain when dealing with large animals, as highlighted by the lack of a reported sequence for *NGN3* in the current pig genome build^41^. To sequence *NGN3*, we used gene synteny to identify conserved sequences both up- and downstream of *NGN3* within human, horse, cow, and dog genomes. Based on these conserved sequences, we designed PCR primers and amplified the pig *NGN3* gene (Deposited into NCBI Genbank; Accession# KP796255) (Fig. 1). Using this genetic information, a single exonic region for *NGN3* was identified for CRISPR targeting and guide RNA design.

### Design and validation of guides

CRISPR guides were generated using MIT software (http://www.genome-engineering.org/ crispr/). Two candidate guides were annealed and transcribed *in vitro* for generation of single sgRNAs for microinjection into embryos, each designed to disrupt the open reading frame (ORF) within an early section of the gene. Mutations early in the ORF are expected to result in frame shifting disruptions within the helix-loop-helix, DNA binding, dimerization interacting domains resulting in a loss of function. A comparison between guides# 1 and 2 using Cas9 protein was performed in cultured embryos (Supplementary Table 1). Guide# 2 injected at 12.5 ng/μL and 25 ng/μL sgRNA and Cas9 protein was ultimately selected based on *in vitro* blastocyst development potential, and induction of mutations from PCR screening analysis for generating *NGN3* null pigs (Supplementary Table 2).

### Microinjection and transfer of *in vivo* derived embryos for generating E60 edited fetuses

A cohort of pigs were estrus synchronized, artificially inseminated, and microinjected with Cas9 ribonucleoproteins targeting *NGN3* (Fig. 1A), and transferred into surrogate animal. The animal confirmed pregnant was euthanized at E60. Table 1 contains data for embryo transfers and efficiencies. A total of 9 fetuses were recovered and imaged (Supplementary Fig. S1). No obvious physiological phenotype was observed in the fetuses at this stage. The fetuses were numbered, and ear and tail samples from respective fetuses were isolated for genotypic, and pancreas for phenotypic analysis. Fetal fibroblast lines were established from all collected fetuses (n = 9). Screening primers for *NGN3* (Supplementary Table 3) were used to amplify the target region from the genomic DNA of each respective animal. Targeting efficiency of CRISPR/Cas9 reagents resulted in 94% (17/18) of alleles being edited. Animals #2, 4, and 6 demonstrated frame shifting mutations leading to the introduction of an early stop codon for both alleles following PCR amplification, cloning and sequencing (Fig. 1B,C; Supplementary Fig. S2). Amino acid sequences of WT and Animal #2 were 33.2% identical, with 40% similarity, and 5% gaps using pairwise alignment (EMBOSS Stretcher feature (www.ebi.ac.uk)) (Fig. 1C). Animals# 1, 3, 5, 8 and 9 demonstrated in-frame mutations (Fig. 1B). Animal# 7 had a single WT allele and a frame-shift mutation on the other allele.

**Table 1:** Generation of CRISPR/Cas9-mediated *NGN3* targeted E60 fetuses.

No. embryos transferred	No. recipients	No. (%) pregnant	No. fetus and (PE%)^*^	# of Edited Fetuses
61	1	1/1 (100)	9 (14.8)	9

^*^Production Efficiency (PE) was calculated as the percentage of the number of fetuses relative to the number of embryos transferred to recipients.

#### Phenotypic analysis of E60 *NGN3* edited fetuses

**Figure 2:**
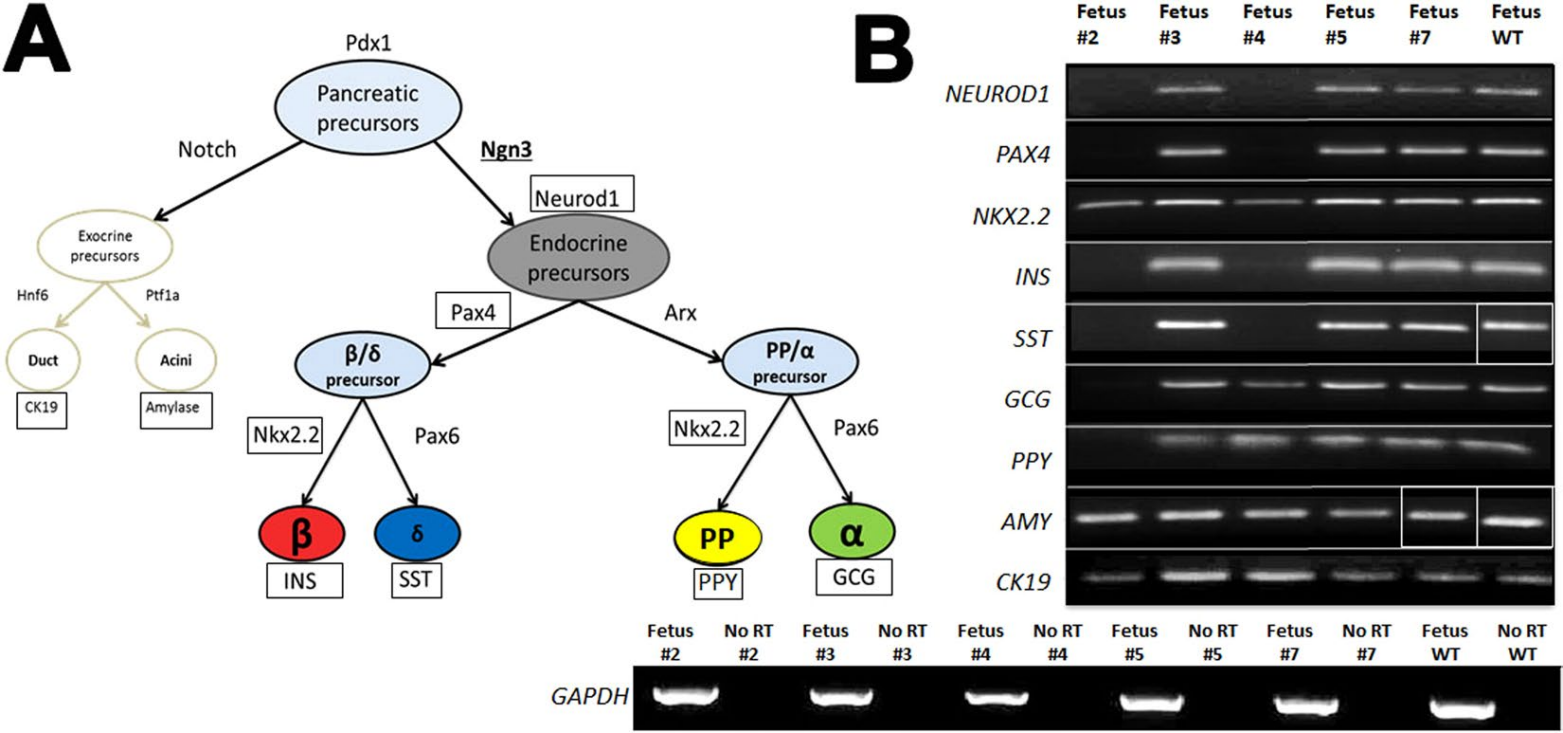
RT-PCR screening for endocrine perturbation in NGN3-edited E60 fetuses. (**A**) Schematic showing exocrine and endocrine lineage specification in the pancreas, and representative marker genes expressed in respective precursor or specialized cell types. (**B**) Combined 2% agarose gel images from amplicons using RT-PCR from pancreas tissue cDNA. Gene-specific primers were used following first strand-synthesis (OligodT) for *NEUROD1, PAX4, NKX2.2, INS, SST, GCG, PPY, AMY, CK19*. Fetuses #2, #3, #4, #5, #7 and WT control were used for representation of pancreas gene profiles following NGN3-gene targeting. Cropped and merged image boundaries are depicted by the white borders.

Total RNA from nine *NGN3*-edited E60 fetal pancreata was isolated. Wild-type E60 fetal pancreas was collected as a control. Based on the genotyping data, we focused primarily on mutant animals with genotypes that introduced frame shifting mutations or early stop codons in predicted amino acid sequence compared to WT (Fetus# 2, 3, 4, 5 and 7). Among the edited piglets, candidate genes that are expressed in the exocrine (CK19 and Amylase) cells and genes downstream of *NGN3* that mark the precursor endodermal cells (*NEUROD1; NKX2.2*, and *PAX4*), and specialized pancreatic cells (*INS, SST, PPY*, and *GCG*) were screended (Fig. 2A; Supplementary Fig. S3). Among the edited piglets, Animal #4 displayed a loss of *NEUROD1*, *PAX4*, *INS*, and *SST*; but maintained expression for *GCG* and *pancreatic polypeptide Y* (*PPY*) transcripts, albeit at lower levels compared to WT animals (Fig. 2B). In Animals #3, 5 and 7, the expression of endocrine and exocrine genes was unperturbed. In Animal #2, however, there was a complete loss of endocrine population as evidenced by the lack of *NEUROD1*, *PAX4* (direct downstream targets of *NGN3*)^42–44^, *INS*, *GCG*, *SST*, and *PPY* transcripts. Consistent with previous data from mouse models, *NGN3* ablation did not perturb exocrine pancreas formation as evidenced by the maintenance of Amylase (*AMY*), and Cytokeratin-19 (*CK-19*) expression (Fig. 2B). In Animal #2 that showed the most striking expected loss of endocrine population, the biallelic 2 bp deletion results in alteration of the ORF and introduction of a premature stop codon (Supplementary Fig. S2). This data support the likelihood for mutations to result in loss of NGN3 function, with a loss of putative downstream targets and endocrine hormones. Animal #6 was not included in the analysis due to RNA degradation and therefore, was not a candidate for SCNT. The other animals had expression profiles equivalent to the wild type controls (Fig. 2).

### Somatic cell nuclear transfer and confirmation of cloning

To determine whether mutations occurring at the NGN3 locus would inhibit full-term gestation, fetal fibroblasts derived from Animal #2 were used as donors for SCNT. Cloned embryos (n = 102) were transferred into a surrogate gilt (Table 2), resulting in a pregnancy. At term, 5 piglets were born, 3 were alive at birth, and 2 were stillborn. These newborns showed a markedly reduced weight within 36 hours after birth requiring humane euthanasia. Genotypes from the clonal pigs were confirmed as biallelic knockouts (Δ2/Δ2) following PCR and sequencing.

**Table 2:** Generation of CRISPR/Cas9-mediated *NGN3*-edited cloned piglets.

No. embryos transferred	No. recipients	No. (%) pregnant	No. piglet and (CE%)*	No. live piglets
102	1	1/1 (100)	5 (5.4)	3/5

Five piglets were naturally delivered on Day 117, and of the 5 piglets born, 3 were alive at birth, 2 were stillborn. The cloning efficiency (CE) was 5.4%, calculated as the percentage of the number of piglets relative to the number of embryos transferred to recipients.

#### Phenotyping SCNT derived clonal *NGN3* null animals

**Figure 3:**
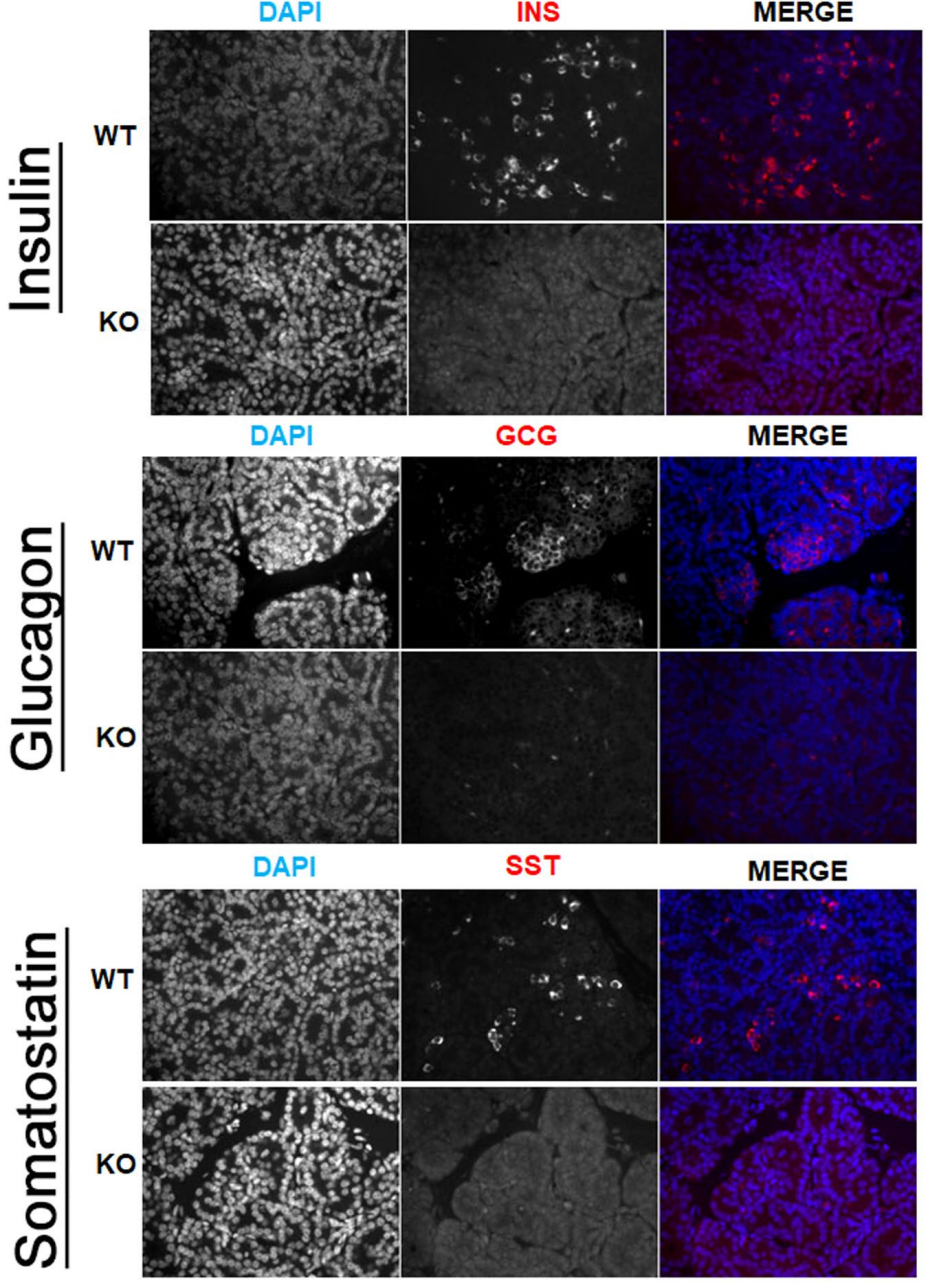
Hormone expression as detected by immunofluorescence on paraffin embedded pancreas tissues from *NGN3* null and newborn WT piglets. Representative hormone expression from newborn WT and NGN3-mutant cloned pancreas. Formalin fixed, paraffin embedded and 5 μm sections of newborn pancreas were stained using Guinea Pig anti-INS (DAKO), anti-GCG, or anti-SST antibodies, and DAPI nuclear stained, and imaged at 40×. Top Row: WT pancreas isolated from newborn piglets, DAPI (Left); INS/GCG/SST in (Middle); and Merge (Right). Bottom Row: *NGN3*-null pancreas isolated between 24–36 hours from newborn piglets. Hormones are pseudo-colored in red. Scale bar 100 μm.

**Figure 4:**
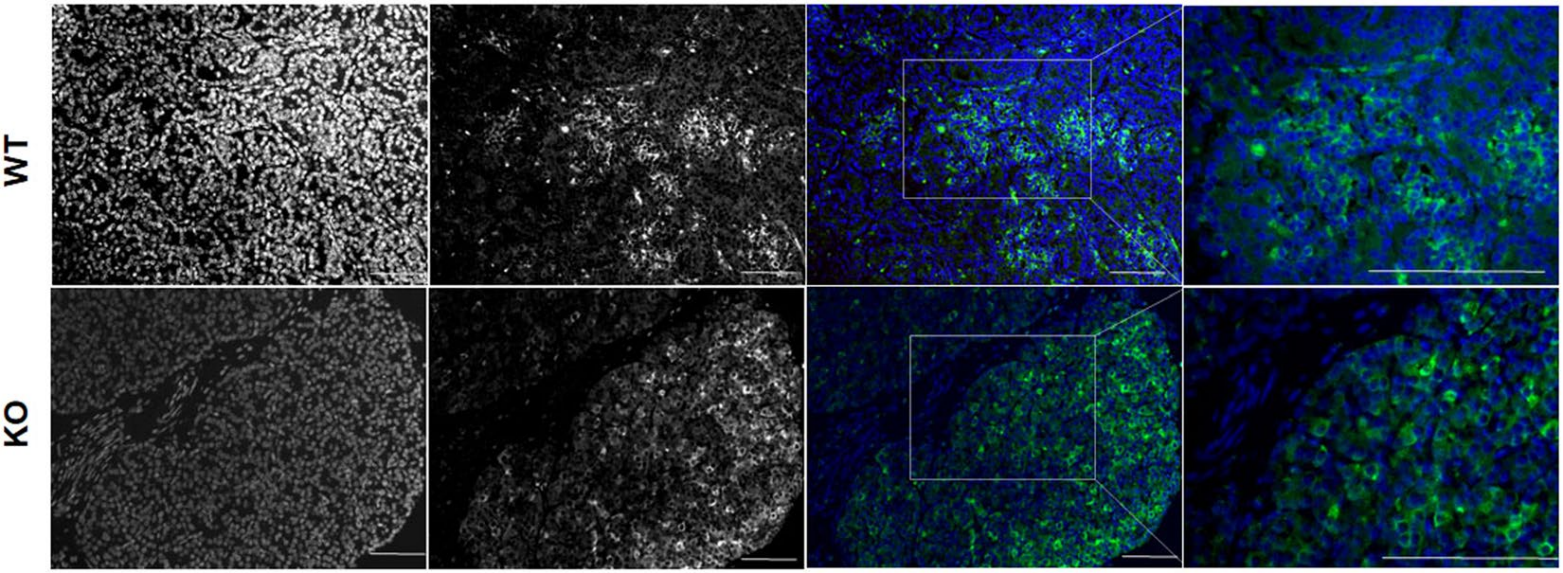
Exocrine AMY expression from WT and *NGN3*-KO pancreas sections. Comparable levels of AMY expression between WT and KO pancreas sections. Top row: DAPI nuclear stain (Blue) overlaid with AMY (Green) expression for WT pancreas at 20× with focal frame (left column) and 40× image of framed area (right column). Bottom row: DAPI nuclear stain (Blue) overlaid with AMY (Green) within cloned NGN3-KO pancreas at 20× with focal frame (left column) and 40× image of framed area (right column). Scale bar 100 μm.

Pancreas samples were stained for INS, GCG, and SST to determine the role of NGN3-editing on endocrine hormone production. Positive staining was observed in all control wild type tissues for INS, GCG, and SST (Supplementary Fig. S4). Cloned NGN3 null pancreas sections demonstrated loss of GCG and SST positive cells upon analysis of sections from each mutant compared to secondary only controls and staining pattern from wild type animals, respectively (Fig. 3). INS was significantly reduced compared to age-matched controls in mutant pancreas sections. Figure 3 illustrates representative staining for INS, yet a more thorough analysis revealed expression in sparse patches in all mutant pancreata (Supplementary Fig. S5). INS and DAPI expression was quantified. Outliers >1.5× the inter-quartile range above the third quartile were removed. Age-matched WT pancreas tissue was used to normalize INS expression. INS expression was 1.85% ± 1.88% across all mutant pancreata. AMY expression was not altered between WT and cloned pancreata (Fig. 4) and upon histological assessment, ductal structures are comparable (Supplementary Fig. 6). These data support a vital role for *NGN3* in the expression of INS, GCG and SST.

## Discussion

*Ngn3* is necessary and sufficient for endocrine pancreas formation in the mouse, as demonstrated by genetic KO and lineage tracing experiments^16,45–47^. Our data support a similar requirement of *NGN3* for endocrine pancreas development in the pig. In our initial targeting experiments investigating E60 fetuses, we established genotyping data supportive of a functional KO for Animal #2. We show decreased expression of critical downstream targets of *NGN3*, and a loss of transcripts for all four endocrine hormones in the pancreas at E60. In Animal #2, absence of *NEUROD1*, *PAX4* and all four endocrine hormones was observed and independently replicated using RT-PCR. In Animal #4, strong signal for NKX2.2, GCG and PPY and a mild signal for insulin was noticed. This was unexpected from the predominant frame shifting mutations from the genotyping analysis. One posssible explanation is the occurance of a minor allelic mosaicism in the fetus, with few cells bearing potential wild type or in-frame deletions that were not detectable from our genotyping results, and thereby contributing to the endocrine population. Animal #6, was another ideal candidate with Δ25/Δ25 biallelic modification. However, degradation of RNA from pancreatic preps precluded initial characterization, and was therefore not used in subsequent trials.

Using the preliminary data from E60 pancreata as an indication of perturbed *NGN3* function, Animal #2 fibroblast lines were used as nuclear donors in a subsequent experiment. Reconstructed cloned embryos were successfully transferred to a single surrogate gilt. Pregnancy was confirmed on D30 of gestation using ultrasound, and allowed to develop to term, resulting in the birth of three live and two still born piglets. All animals were confirmed to be clones of the parent fibroblast line with a biallelic Δ2/Δ2 in-frame deletion. Homozygous clones were also assessed for expression of mature hormones. Like *Ngn3*-null mice^45^, which are indistinguishable from wild type pups, cloned pigs demonstrated similar appearance in size and mass to wild-type clones at birth. *Ngn3*-null mice fed normally for 48–72 hours, while *Ngn3*-null pigs fed normally for 24–36 hours. Mice lost a significant amount of weight over 48 hours, while the cloned piglets demonstrated significant reduction in body mass within 24–36 hours (data not shown). Mice with an ablation of *Ngn3* died within 72 hours, while *NGN3* knock-out pigs required humane euthanasia within 36 hours due to severe reduction in body mass and body condition^45^. These correlations support a conserved role for *NGN3* across species. Our data demonstrate a critical role for *NGN3* in the developing and newborn pig pancreas, specifically endocrine hormone expression. We show a loss or a significant reduction in mature hormone expression, most notable by a loss of GCG and SST expression, and an approximate 98% reduction in INS in the pancreas from newborn cloned mutant piglets.

In the current study, we established a significant role for *NGN3* in the formation of the developing and cloned-neonatal endocrine pancreas, and respective hormone-producing cells types, based on RT-PCR and immunostaining, in the domestic pig. In the vast majority of cells within the pancreas of newborn mutant animals, no expression of INS was detected (~98% INS negative). In about 2% of cells, INS protein was detectable in sparcely dispersed patches. INS expression levels were normalized to WT-control newborn piglets. This data is intriguing, and raise interesting questions regarding endocrine development in the pig. It is reasonable to suggest the role of alternative NGN3- independent compensatory signaling pathways directing INS production in newborn piglets, but further investigation is required. Temporal expression of *NGN3* in human pancreas development differs from the biphasic expression pattern seen in the mouse^29^, which may be more similar to pig pancreas development, further driving the need for additional investigation into pig pancreas development.

There is a greater interest in the porcine model for xenotransplantation and more importantly as a source for generating human-pig chimeras for generating human organs in the pig model^48^. Several recent publications in which *PDX1* was ablated resulted in pancreatic agenesis and have been employed as a means for facilitating organogenesis from donor embryos, paving the way for human stem cell or tissue specific stem cell mediated organogenesis^36,49,50^. This manuscript besides investigating the role of *NGN3* in endocrine pancreas development, offers yet another tool kit in facilitating generation of human islets (endocrine cells) within *NGN3*-ablated pigs without perturbation of exocrine pancreas function.

## Materials and Methods

### Sequencing of *NGN3* in pig genome

Using interspecies genetic synteny and sequence homology, PCR primers were designed to amplify the *NGN3* gene from pig genomic DNA (gDNA). Using Phusion DNA polymerase (NEB, Ipswich, MA), primers NGN3h-F, 5′-GCCTGCAGCTCAGCTGAACTT-3′, and NGN3c-R, 5′-AGCCAGAGGCAGGAGGAACAA-3′, were used to amplify a 1024 bp product by PCR using gDNA isolated from a mixed breed pig (Poland China/Large White/Landrace). The PCR amplicon was then A-tailed with 2× OneTaq master mix (New England BioLabs), ligated into the TA cloning vector pCR2.1 (Invitrogen, Carlsbad, California), and transformed into competent NEB5α *E. coli* (New England BioLabs). Plasmids were isolated from transformants using Qiagen Plasmid DNA miniprep kit (Qiagen, Venlo, The Netherlands), and sent to Macrogen USA (Rockville, MD) for sequencing with M13F and M13R primers. Nucleotide BLAST (ncbi.nlm.nih.gov) analysis of the sequencing data confirmed the pig *NGN3* gene. Amplicons from twelve independent PCR reactions were cloned into the pCR2.1 vector and sequenced. Sequencing reads for the *NGN3* CDS were assembled using the Staden package, version 1.7. A 645 bp fragment corresponding to a single exon was deposited into NCBI Genbank; accession KP796255.

### Production and validation of CRISPR sgRNAs

Guides targeting exon 1 of *NGN3* were designed using software from MIT (http://www.genome-engineering.org/crispr/crispr.mit.edu). Guides were prepared as previously described^40^ and eluted in RNase-free water for embryo injections. *In vitro* transcribed sgRNA was quantified using a Nanodrop™ and integrity analyzed using the Agilent Bioanalyzer 2100™. Functionality of sgRNA was validated according to Supplementary Table 1 by injecting 12.5 ng/μL of purified sgRNA along with 25 ng/μL Cas 9 protein (PNA Bio, CA) respectively into parthenogenetically derived porcine embryos, using a FemtoJet microinjector (Eppendorf; Hamburg, Germany). The microinjected parthenogenetic embryos were cultured to blastocyst stage in PZM3 medium for 144 h at 38.5 °C, 5% CO_2_, 5% O_2_ and 100% humidity for screening. Blastocysts were counted and collected for screening of targeting events using PCR and cloned into pCR2.1 (Invitrogen) for sequencing to validate mutational efficiency. A single verified sgRNA was incubated with Cas9 protein to form the ribonucleoprotein complex for subsequent microinjection into *in vivo* matured oocytes (Guide Sequence for embryo microinjection, sgRNA-2: GCTGTTCCCCGGTACGCGAG TGG).

### Collection and microinjection of porcine *in vivo* zygotes with Cas9 ribonucleoproteins and embryo transfers to generate *NGN3* null animals

All experiments involving live animals were performed in accordance with the approved guidelines and regulations of the Beltsville ARS Institutional Animal Care and Use Committee (IACUC). All experimental protocols involving live animals were approved by the IACUC committee. Pubertal gilts were estrus synchronized using Alternogest (Regumate or Matrix, Merck), and checked daily for the onset of estrus (Day 0)^51^. Gilts displaying estrus were inseminated with extended boar semen (Progenes). Zygotes were recovered surgically 24 h later by mid-ventral laparotomy. Briefly, gilts were anesthetized with an intravenous injection of telazol, ketamine, xylazine mixture, and anesthesia was maintained under 5% isofluorane inhalation. The reproductive tract was exposed, and the oviducts were flushed with 25 ml of Hepes-buffered medium. The recovered medium was examined under a dissecting microscope to collect the embryos. All recovered *in vivo* zygotes were immediately placed into a drop of TL-Hepes medium and used for microinjection. A single verified guide with high targeting efficiency was chosen for further experiments. *In vivo* fertilized zygotes were microinjected with a precomplexed *NGN3*-ribonucleoprotein and 61 embryos were surgically transferred into the oviducts of synchronized gilts on the first day of standing estrus. Pregnancies were confirmed by ultrasound around day 30 following transfer. The pregnant sow was euthanized on day 60 of gestation and 9 fetuses were collected for establishment of fetal fibroblast cell lines.

### Somatic cell nuclear transfer (SCNT) to establish clonal NGN3 knockout pigs

Fetal fibroblasts harvested from day 60 fetuses were genotyped (see below). The fibroblast line from Fetus #2 was utilized for SCNT to establish clonal lines of *NGN3* knock-out pigs as described in previous studies^40,52^. One hundred and two cloned embryos were surgically transferred into the oviducts of synchronized gilts on the first day of standing estrus. Pregnancies were confirmed by ultrasound on day 29 following transfer. Parturition of the piglets was induced on day 116 following an injection of oxytocin.

### Genomic DNA isolation and genotyping of edited embryos, fetuses and animals

*Single* blastocysts cultured for 144 h *in vitro* were washed three times with PBS-PVA (pH 7.4) medium and transferred into 9 µl of blastocyst lysis buffer (50 mM KCl, 1.5 mM MgCl_2_, 10 mM Tris pH 8.0, 0.5% NP-40, 0.5% Tween-20 and 100 µg/ml proteinase K) and incubated for 1 h at 65 °C. The digestion was terminated by heating the mixture at 95 °C for 10 min, and 2 µl of supernatant used as a PCR template. Tissue biopsies (ear notch and tail dock) from fetuses and offspring were digested in a tissue lysis buffer (50 mM Tris pH 8.0, 0.1 M NaCl, 20 mM EDTA, 1% SDS, 50 µg/ml RNase A, 100 µg/ml proteinase K) overnight at 65 °C. Following overnight digest, gDNA was extracted using phenol-chloroform, and recovered by resuspension in 100 µl of 10 mM Tris- HCl, pH 7.4 buffer following ethanol precipitation. Purified gDNA was PCR amplified using KOD 2× PCR Master Mix (TOYOBO) over 35 cycles across *NGN3*/CRISPR targeting site (Forward: 5′-CACCAGACCGAGCAGTCTTT-3′ Reverse: 3′-TTGGTGAGTTTCGCATCGT-5′). Resulting products were separated using standard agarose gel electrophoresis and capillary electrophoresis using a Fragment Analyzer™(Advanced Analytical). Products were purified over QIAprep DNA miniprep columns, subject to A-tailing and ligated into pCR™2.1 vector, and transformed into DH5α electrocompetent cells (prepared in-house). Plasmids were isolated from five to fifteen colonies using QIAprep spin miniprep kit, and DNA concentration determined using a NanoDrop ND-1000 spectrophotometer, followed by EcoRI restriction digest to confirm amplicon insertion. An average of 10 colonies were used to confirm genotypes from both the ear and tail PCR amplicons. All sequences were aligned to wild type *NGN3* using ClustalW available though BioEdit software. Mutational analysis was confirmed using NCBI BLAST. Further mutational analysis was performed using translated nucleotide function from NCBI and confirmed using NCBI BLAST functions for identification and localization of early termination sites and altered reading frame from predicted amino acid sequences (Fig. 1).

### Gene expression analysis using RT-PCR

Pancreas tissue was isolated from fetal pigs at day 60 d.p.c. and immediately placed into RNALater Solution (Ambion). Approximately 30 mg of tissue was lysed using Qiagen buffer RLT with Betamercaptoethanol (BME) and polytron benchtop tissue homogenizer. RNA was isolated using a combination of Phase-lock gel tubes and the RNeasy miniprep kit (Qiagen). Synthesis of cDNA was performed using oligo (dT) primers and standard reverse transcription reaction using SuperScript® IV (Invitrogen). 30 cycles of PCR was used to amplify target transcripts along with “No-RT” controls, then run on 2% agarose gel alongside *GAPDH*-amplified cDNA controls using electrophoresis. A gDNA template was used as a control to verify exon spanning amplified transcripts. Amplicon size was confirmed for each target gene. Primer sequences are listed in Supplementary Table 3.

#### Immunostaining

Representative samples from the pancreas at euthanasia were formalin fixed. Fixed pancreata were paraffin-embedded by American Histolabs Inc., Gaithersburg, MD. Immunostaining was performed on 5 µm sections affixed to charged slides and subjected to heat-induced antigen retrieval at 95 °C for 20 minutes in Citrate Buffer at pH 6.0, cooled at room temperature for 30 minutes and rinsed in Phosphate-buffered saline containing 0.01% Triton X-100 (PBST), blocked using 5% Donkey Serum (Jackson Immunoresearch), and incubated overnight at 4 °C in primary antibodies: INS diluted 1:150 (Dako, A0564); GCG 1:200 (abcam, ab10988); SST diluted 1:300 (Dako, A0566) and AMY diluted 1:300 (Cell Signaling Technology, 3796). On the next day, sections were washed in PBST 3 × 10 minutes and incubated with fluorescence labelled secondary antibody diluted in PBST: Alexa Fluor Donkey 488 (1:1500), and/or Alexa Fluor 568 (1:1500) against primary antibody host species for 30 minutes in the dark at room temperature. The slides were mounted with Prolong-Gold Antifade Mount with DAPI (Life Technologies), staining the nuclei. Negative control slides were included in each experiment by excluding the respective primary antibodies. Imaging was performed using an upright fluorescent microscope with an automated stage (Nikon Eclipse Ni-U).

## Electronic supplementary material

Supplementary Information
