# Supplementary material for: Targeted Mutation of NGN3 Gene Disrupts Pancreatic Endocrine Cell Development in Pigs
Source: Sci Rep. 2018 Feb 26;8:3582. doi: 10.1038/s41598-018-22050-0 (PMC5827570; doi:10.1038/s41598-018-22050-0)
Supplement: Supplementary file 1 — Supplementary Information [file 41598_2018_22050_MOESM1_ESM.docx]

Supplementary Data

**Targeted Mutation of *NGN3* Gene Disrupts Pancreatic Endocrine Cell Development in Pigs**

Timothy P. Sheets^1,2^, Ki-Eun Park^1,2^, Chi-Hun Park^1,2^, Steven M. Swift^1,2^, Anne Powell^2^, David M. Donovan^2^, Bhanu P. Telugu^1,2,3*^

^1 Department of Animal and Avian Sciences, University of Maryland, College Park, MD 20742, USA^

^2 Animal Bioscience and Biotechnology Laboratory, USDA-ARS, Beltsville, MD 20705, USA^

^3 RenOVAte Biosciences Inc, Reisterstown, MD 21136, USA^

***** Correspondence: btelugu@umd.edu; Tel.: +01-573-529-3852

**Supplementary Table 1**. Verification of *in vitro* guides co-microinjected with Cas9 mRNA or Cas9 protein.

| *NGN3* sgRNA | Cas9 mRNA or Protein | Injected Embryos | Blastocyst Total (%) | Expanded Blastocyst (%) | No. of Expanded Blastocysts Screened | Targeting efficiency | |
| --- | --- | --- | --- | --- | --- | --- | --- |
| Guide 1 | Protein | 50 | 26(52) | 12(24) | 8 | | 67% |
| Guide 1 | mRNA | 50 | 22(44) | 10(20) | 5 | | 50% |
| Guide 2 | Protein | 50 | 24(48) | 16(32) | 12 | | 75% |
| Guide 2 | mRNA | 50 | 24(48) | 12(24) | 7 | | 58% |

Guides #1 or #2 were co-injected with either Cas9 mRNA or Cas9 protein. Effects on embryo development were assessed through the number of developed blastocysts compared to total number of zygotes injected, expanded blastocysts compared to total number of zygotes injected; developmental efficiency; number of blastocysts edited; and targeting efficiency.

*Targeting efficiencies were calculated based on the number of blastocyst stage embryos edited divided by the number of blastocysts/expanded blastocysts screened. Editing events were determined using PCR and sequencing.

**Supplementary Table 2**. *In vitro* testing using guide #2 and Cas9 protein.

| sgRNA3 *NGN3* (ng) | Cas9 Protein (ng) | Injected Embryos | Blastocyst  Total (%) | Expanded Blastocysts(%) | Targeting Efficiency |
| --- | --- | --- | --- | --- | --- |
| 12.5 | 25 | 86 | 44 (51) | 28 (33) | 75% |

A total of 33 embryos were injected with 12.5ng sgRNA and 25ng Cas9 protein. Over 6 days following injection, 6 embryos developed to blastocyst stage, 11 were expanded blastocysts, yielding a developmental efficiency of 52% (17/33). Upon sequence analysis of 12 out of 17 blastocysts or expanded blastocyst stage embryos, 3 injected embryos maintained wild type alleles, while 9/12 blastocysts were mutants resulting in a targeting efficiency of 75%.

**Supplementary Table 3**. RT-PCR primer sequences used to amplify cDNA prepared using RNA isolated from E60 fetal pancreas.

| RT-PCR Primer Sequences | | |
| --- | --- | --- |
| *GAPDH* | Forward  Reverse | TGGCAAAGTGGACATTGTCG  CCCTGTTGCTGTAGCCAAATTC |
| *NKX2.2* | Forward | AAACCCCTTCTACGACAGCAG |
|  | Reverse | ACTTGGAACTCGAGTCTTGGG |
| *NEUROD1* | Forward | AGACGAATGAAAGCCAACGC |
|  | Reverse | TTTTGGCTAAGCGCAGTGTC |
| *PAX4* | Forward | GAATCCTTGGGGCTCTCCAG |
|  | Reverse | AGCCAAGCCAGAGCAAGG |
| *INS* | Forward | ATGGCCCTGTGACCTGGTG |
|  | Reverse | CTTGGGCGTGTAGAAGAAGC |
| *GCG* | Forward | TTGTGCAGTGGCTGATGAAC |
|  | Reverse | TGAATTCCTTGGCAGCTTGG |
| *SST* | Forward | TCCGTCAGTTTCTGCAGAAGTC |
|  | Reverse | TTCTCTGTCTGGTTGGGTTCAG |
| *PPY* | Forward | AAGTGCAGCTCCTTACATGC |
|  | Reverse | AGAAGCAGGAGGTGGCATTAC |
| *AMY* | Forward | TGGGACGCTAGACTGTACAAAG |
|  | Reverse | ATTTCTTGCCCAACGGTAGC |
| *CK19* | Forward | AGAACCACGAGGAGGAAATCAG |
|  | Reverse | TCTCATGTCGCTCAGGATCTTG |

**Supplementary Figure.S1**


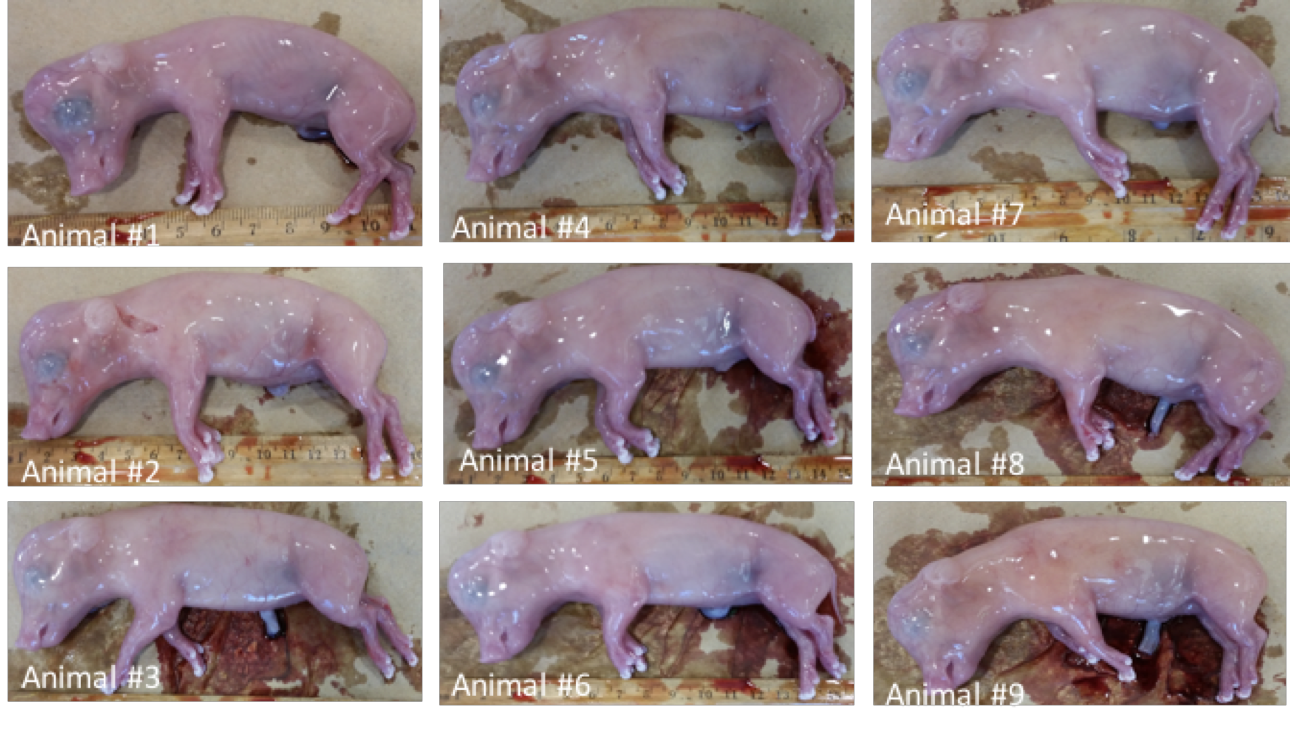
 **Supplementary Fig 1.** Embryonic day 60 fetuses generated through microinjection of *NGN3* CRISPR-reagents and embryo transfer. Nine embryonic day 60 fetuses resulting from the first round of mutational screening. *In vivo*–derived embryos were microinjected with a ribonucleoprotein complex (*NGN3*-sgRNA + Cas9) resulting in a gene-editing efficiency of 94%. No overt phenotypes were identified at this time point.

**Supplementary Figure.S2**

**Wild type:**

**1 ATGGCGCCTC ATCCTTCGTG TGCGCCAGCT GTCCAAGTGA CCCACCAGAC**

**M A P H P S C A P A V Q V T H Q T**

**51 CGAGCAGTCT TTCCCGAGCG CCCCAGAAGA CAAAGTGACC TGCGTCGCAT**

**E Q S F P S A P E D K V T C V A S**

**101 CCGCTCCGCC CAGCCCCACT CGCGTACCGG GGAACAGCGC GGAAGCGGAA**

**A P P S P T R V P G N S A E A E**

**151 GGGGGTGGCT GCCGAGCGGC CTCGAGGAAG CTCCGGGCGC GGCGTGGGGG**

**G G G C R A A S R K L R A R R G G**

**201 GCGCAGCCGG CCCAAGAGTG AGTTGGCTCT GAGCAAGCAG CGACGGAGCC**

**R S R P K S E L A L S K Q R R S R**

**251 GGCGCAAGAA GGCCAACGAC CGTGAGCGCA ATCGAATGCA CAATCTCAAC**

**R K K A N D R E R N R M H N L N**

**301 TCCGCGTTGG ATGCGCTGCG CGGGGTCCTG CCCACCTTCC CGGACGATGC**

**S A L D A L R G V L P T F P D D A**

**351 GAAACTCACC AAGATAGAGA CGCTACGCTT CGCACACAAT TACATCTGGG**

**K L T K I E T L R F A H N Y I W A**

**401 CGCTGACGCA AACGCTGCGC ATAGCGGACC ACAGCCTCTA CGGGCTGGAG**

**L T Q T L R I A D H S L Y G L E**

**451 CCGCTTGCAC CGACCTGCGA GGAGCTGGGC AGCCCGGACG GCTCCCCGGG**

**P L A P T C E E L G S P D G S P G**

**501 AGACTGGGGC TCCCTTTATT CCCCAGTCTC CCAGGCGGGC AGCTTGAGCC**

**D W G S L Y S P V S Q A G S L S P**

**551 CCGTTGCCTC GCTGGAGGAG CGCCCTGGGC TGCAGGCGCC TGCGTCCCCT**

**V A S L E E R P G L Q A P A S P**

**601 GCCTGCTTGC ATCCTGGCGC CCTGGCCTTT TCAGACTTTC TATGA**

**A C L H P G A L A F S D F L ***

**Animal #2 (Δ2/ Δ2)**

**1 ATGGCGCCTC ATCCTTCGTG TGCGCCAGCT GTCCAAGTGA CCCACCAGAC**

**M A P H P S C A P A V Q V T H Q T**

**51 CGAGCAGTCT TTCCCGAGCG CCCCAGAAGA CAAAGTGACC TGCGTCGCAT**

**E Q S F P S A P E D K V T C V A S**

**101 CCGCTCCGCC CAGCCCCACT CGTACCGGGG AACAGCGCGG AAGCGGAAGG**

**A P P S P T R T G E Q R G S G R**

**151 GGGTGGCTGC CGAGCGGCCT CGAGGAAGCT CCGGGCGCGG CGTGGGGGGC**

**G W L P S G L E E A P G A A W G A**

**201 GCAGCCGGCC CAAGAGTGAG TTGGCTCTGA GCAAGCAGCG ACGGAGCCGG**

**Q P A Q E ***

**251 CGCAAGAAGG CCAACGACCG TGAGCGCAAT CGAATGCACA ATCTCAACTC**

**301 CGCGTTGGAT GCGCTGCGCG GGGTCCTGCC CACCTTCCCG GACGATGCGA**

**351 AACTCACCAA GATAGAGACG CTACGCTTCG CACACAATTA CATCTGGGCG**

**401 CTGACGCAAA CGCTGCGCAT AGCGGACCAC AGCCTCTACG GGCTGGAGCC**

**451 GCTTGCACCG ACCTGCGAGG AGCTGGGCAG CCCGGACGGC TCCCCGGGAG**

**501 ACTGGGGCTC CCTTTATTCC CCAGTCTCCC AGGCGGGCAG CTTGAGCCCC**

**551 GTTGCCTCGC TGGAGGAGCG CCCTGGGCTG CAGGCGCCTG CGTCCCCTGC**

**601 CTGCTTGCAT CCTGGCGCCC TGGCCTTTTC AGACTTTCTA TGA**

**Supplementary Fig 2**. Sequence of wild type NGN3 coding sequence and mutated sequence in Animal#2. In the figure, putative guide sequence for Cas9 is highlighted in yellow, and PAM motif (TGG) in green. The guide pairs to sequence in the opposite strand. The predicted Cas9 cut site is shown as a ( ). In Animal #2, the two nucleotides adjacent to the cut site (CG) were deleted resulting in frame shift (shown in red) and premature stop codon (*) in the open reading frame, resulting in the loss of protein function.

**Supplementary Figure. S3**

**
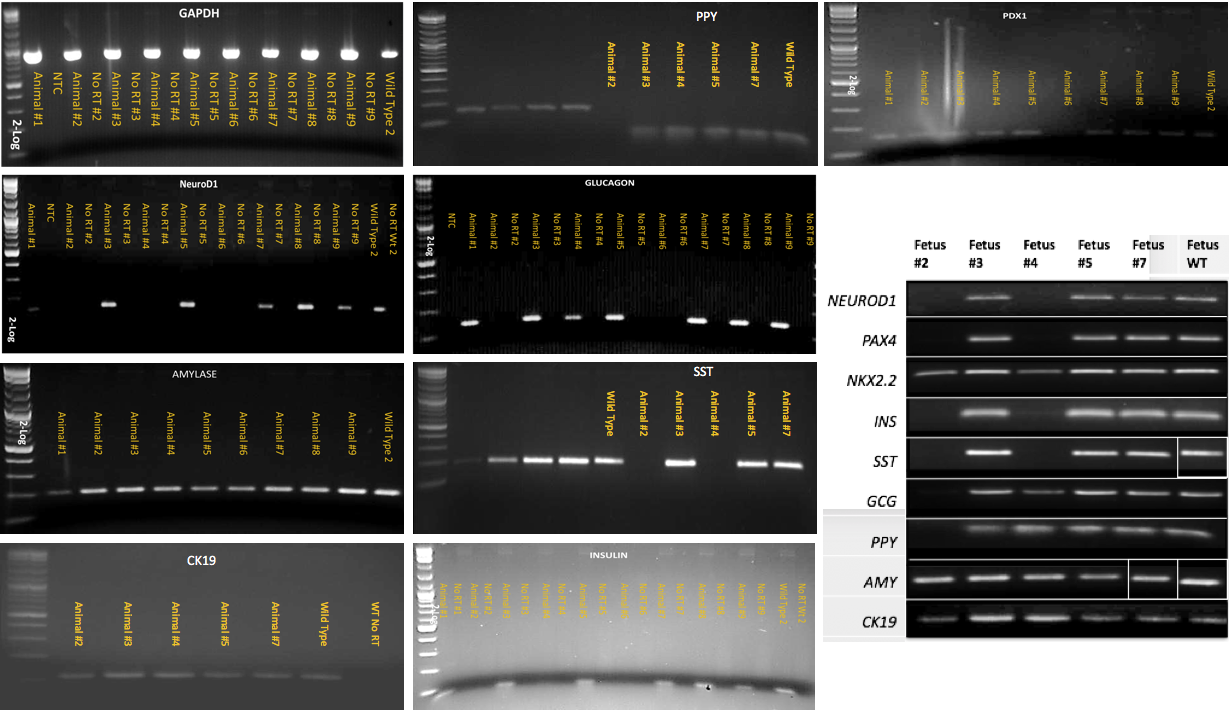
**

**Supplementary Fig.3**. Uncroped RT-PCR images run on 2% agarose gels as represented in Fig. 2. Transcripts and sample identifiers are presented from left to right, and top to bottom for: GAPDH, PPY, NEUROD1, Glucagon, Amylase, SST, CK19, and Insulin.

**Supplementary Figure. S4**


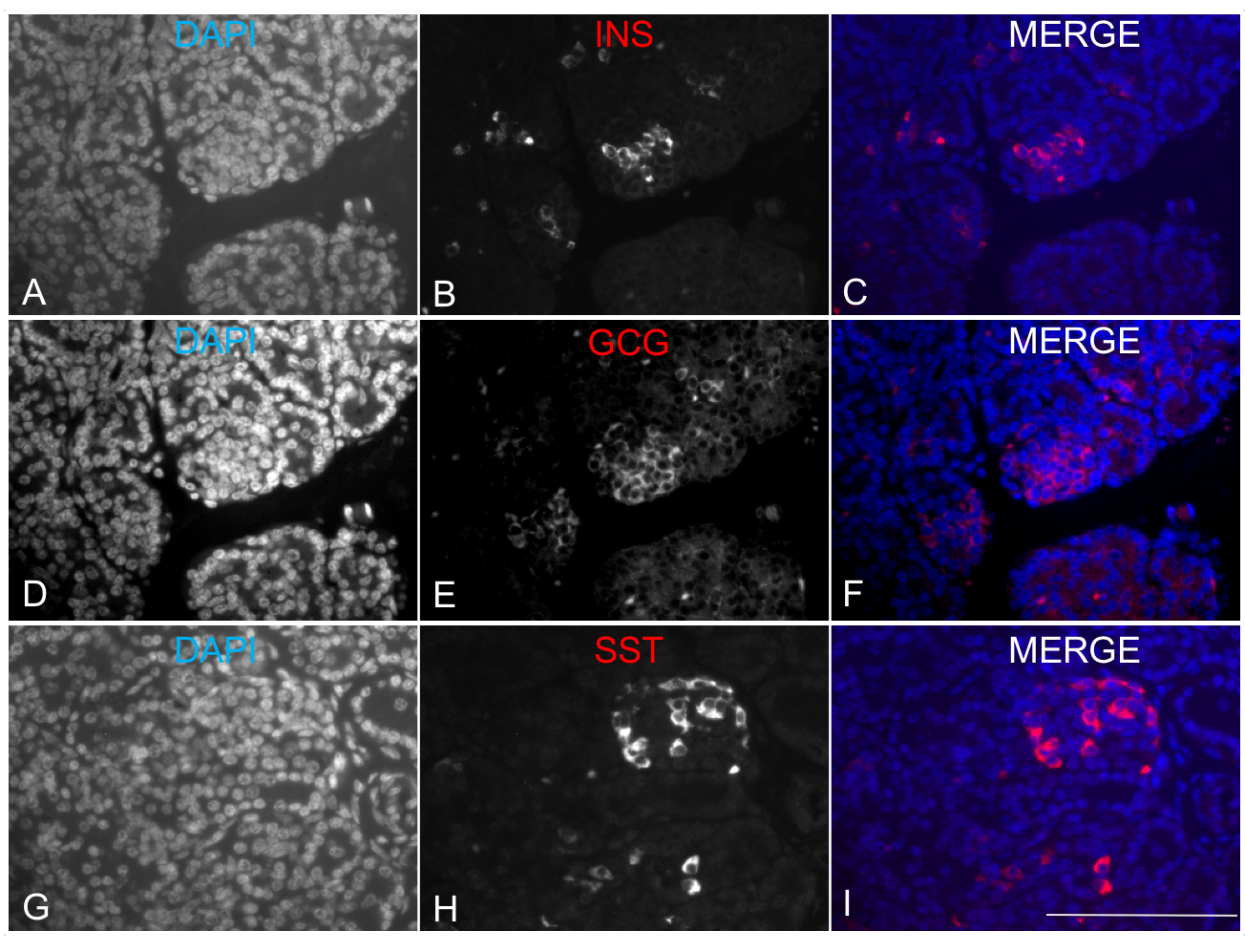


**Supplementary Fig 4**. Hormone expression as detected by immunofluorescence on paraffin embedded pancreas tissues from newborn WT piglets. (A) DAPI staining (B) INS (C) Merge; (D) DAPI (E) GCG (F) Merge; (G) DAPI (H) SST (I) Merge. Images A-C and D-F were co-stained on the same section with INS and GCG, images captured using different exposures and hormones pseudocolored red. All images taken at 40X magnification. Scale bar 100μm.

**Supplementary Figure. S5**


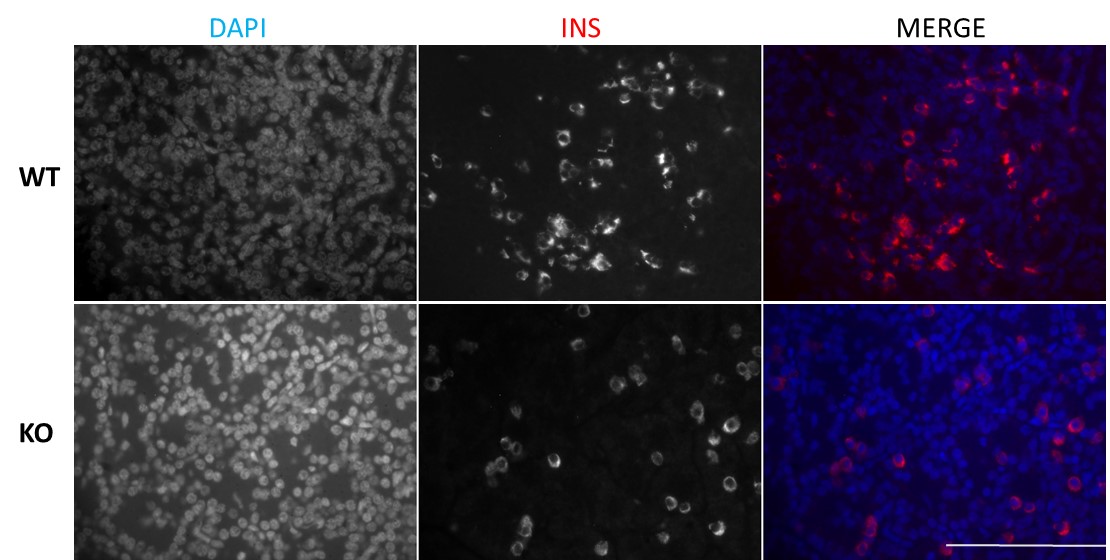


**Supplementary Fig 5**. INS expression in WT and rare INS expression detected in ~2% of cells within the cloned, NGN3-mutant pancreas. Cloned NGN3-mutant animals rare expression of insulin in the pancreas as detected by immunofluorescence in paraffin embedded tissues from newborn piglets. WT pancreas: INS expression is represented in the WT pancreas in red (Top Row). Cloned NGN3-mutant pancreas expressed INS (red) in sparse patches throughout ~2% tissue compared to the WT. DAPI nuclear staining (blue). Scale bar 100μm.

**Supplementary Figure. S6**


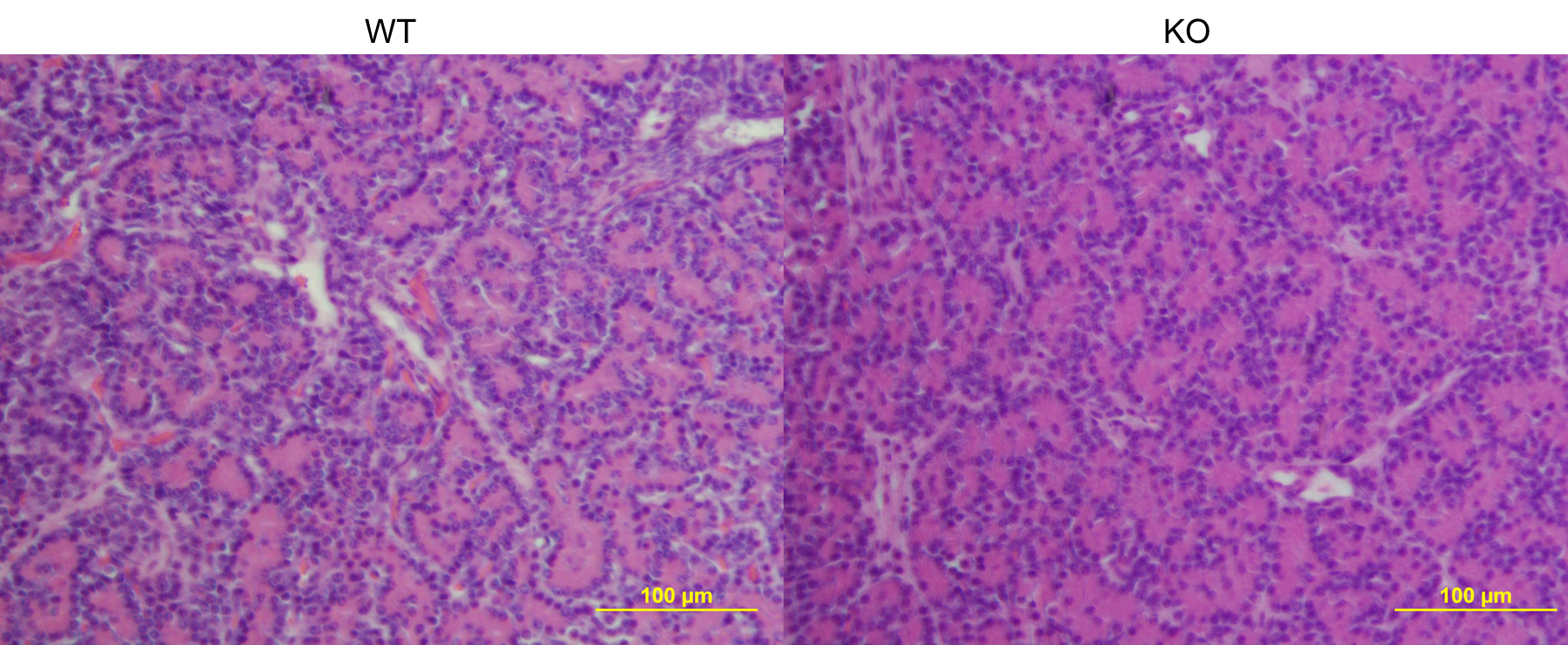


**Supplementary Figure 6**. H&E stain demonstrating similar morphology between WT and *NGN3*-KO pancreas sections. H&E as a morphological representation of pancreas tissue between WT and KO newborn piglets. WT pancreas section (right column), KO pancreas section (left Column). Images taken at 20X magnification.
